# Fat soluble vitamin levels in children with newly diagnosed celiac disease, a case control study

**DOI:** 10.1186/s12887-018-1107-x

**Published:** 2018-04-09

**Authors:** Yavuz Tokgöz, Semiha Terlemez, Aslıhan Karul

**Affiliations:** 1 Department of Pediatric Gastroenterology, Hepatology and Nutrition, Adnan Menderes University Medical Faculty, 09100 Aydın, Turkey; 2 Department of Pediatrics, Adnan Menderes University Medical Faculty, Aydın, Turkey; 3 Department of Biochemistry, Adnan Menderes University Medical Faculty, Aydın, Turkey

**Keywords:** Celiac disease, Children, Fat-soluble vitamins

## Abstract

**Background:**

In children diagnosed with celiac disease, fat soluble vitamin levels were aimed to be evaluated and it was intended to determine whether fat soluble vitamin levels were needed to be assessed routinely in these patients during diagnosis.

**Methods:**

Between May 2015–May 2016, diagnosis symptoms of celiac patients (CD) in newly diagnosed pediatric group were questioned, fat soluble vitamin levels simultaneous with intestinal biopsies were evaluated. Vitamin levels were compared with those of healthy control group.

**Results:**

A total of 52 patients involving 27 female (51.9%), 25 male (48.1%); and a total of 50 healthy control group including 25 female (50%), 25 male (50%) were evaluated. The average age of patients was 9 ± 4.3 years, and their average weight was determined as 16.2 ± 6.3 kg. Growth retardation was the most frequent symptom in our patients (61.5%). Abdominal pain (51.9%) and diarrhea (11.5%) are among the other most commonly seen symptoms. In the histological examination of patients, Marsh 3B *n* = 23 (45.1%) was mostly established. Vitamin A and vitamin D levels of patients were determined significantly lower compared to those of control group. Vitamin A and vitamin D deficiencies were identified significantly higher compared to those of healthy control group. Vitamin D insufficiency was observed in 48 patients (92.3%) and vitamin D deficiency was determined in 32 (61.5%) out of 48. Vitamin A deficiency was established in 17 (32.7%) patients. Vitamin E and vitamin K1 deficiency were determined in no patients. In the healthy control group, vitamin D deficiency was seen in 2 (4%) patients, vitamin D insufficiency was determined in 9 (18%) patients. Other vitamin levels were identified at normal levels in the healthy group.

**Conclusions:**

In newly diagnosed children with CD, a significant lowness was established in vitamin D and A. The evaluation of vitamin A and D levels will be helpful in the course of diagnosis in these patients.

## Background

Celiac disease (CD) is malabsorption syndrome appearing as a result of intolerance developed against gluten found in cereals such as barley, rye and oat and cereal proteins similar to gluten in individuals genetically susceptible. It is thought that the disease develops with autoimmune mechanisms in genetic base [1–3]. The disease has been initiated to be seen at an increasing rate and therefore more information has been added to its clinical features. While the clinical findings of celiac disease are variable to a large extent, the most noteworthy symptoms emerge due to proximal intestinal malabsorption. In accordance with gluten amount and immunological response of an individual, malabsorption findings can appear in weeks or months [4–6]. Vitamin and mineral deficiencies developed secondary to malabsorption cause numerous clinical findings to emerge in patients. Most commonly known condition in celias disease is vitamin D deficiency [7–10]. Osteoporosis and hyperparathyroidism findings of patients have been linked to vitamin D deficiency [7, 11]. The other vitamin deficiency leading to crucial clinical findings is vitamin K in celiac patients. Some parts of hemostasis disorders developing in patients are related to vitamin K insufficiency [12]. The routine control of fat soluble vitamin levels is recommended in patients who have been diagnosed with celiac disease at adult ages [13, 14]. Nevertheless, there is no agreement on vitamin supplement application or the routine evaluation of fat soluble vitamins in patients diagnosed at a young age. Our study aimed to evaluate fat soluble vitamin deficiencies during diagnosis of children who have been recently diagnosed with celiac disease and determine whether fat soluble vitamin levels are needed to be assessed during the diagnosis of these patients.

## Methods

### Patients

Pediatric patients aged between 0 and 18 and diagnosed with CD between May 2015–May 2016 by Adnan Menderes University Medicine Faculty Children’s Gastroenterology Department were included in the study. The diagnosis of celiac disease was made either with anti-endomysial IgA level or tissue transglutaminase IgA levels. In addition, it was confirmed by endoscopic biopsy. Weight, height, body mass indices (BMI) of patients and their physical examination findings were evaluated. In addition, the probable indications associated with symptoms (rash, bleeding, easy bruising and etc.) and vitamin insufficiencies of patients during their reference to hospital were questioned. Hematologic evaluations together with vitamin levels during the diagnosis of patients, tissue transglutaminase levels and albumin levels were also reviewed. Histological categorization was performed with endoscopic biopsy materials. The demographical, hematologic data and fat soluble vitamin levels of patients were compared with those of healthy control group with similar age and numbers. To reduce the differences to minimum that seasons can form for vitamin D level, season distribution was chosen to be similar between patient group and control group.

### The evaluation of fat soluble vitamin levels

After patients’ blood samples had been obtained and their serum part separated, they were kept at -80^o^ C until they were analyzed. Vitamin D levels were evaluated with Chemiluminescence method in Architect hormone autoanalyzer (C8000 Architect, Abbott, Abbott Park, IL, U.S.A). In patients whose vitamin D level was < 30 ng/ml vitamin D insufficiency; in those whose vitamin D level was < 20 ng/ml vitamin D deficiency was defined [15]. Vitamin E, K1 and vitamin A levels were evaluated with ELİSA method. The method of ELİSA kits in question is based on competitive enzyme immunoassay principle.

The results attained were assessed in accordance with most commonly used normal intervals of vitamin levels in literature [15] (Table 1).

**Table 1:** The fat-soluble vitamin-level reference value [15]

Vitamin D (ng/mL)	Deficiency	< 20
	Insufficiency	< 30
Vitamin A (ng/mL)	1–6 age	2–4,3
	7–12 age	2,6–4,9
	13–19 age	2,6–7,2
Vitamin E (mcg/mL)	1–12 age	3–9
	13–19 age	6–10
Vitamin K1 (pg/ml)		100–2200

### Histological evaluation

Though Marsh criteria have been taken as a basis for histopathological diagnosis, it is also recommended to make some changes. We used these formed criteria with the development of Marsh criteria in the histological categorization [16]. According to this classification;

- Type 1: Normal villus structure together with increased intraepithelial lymphocyte (IEL).
- Type 2: Shortened villus structure together with IEL and crypt hyperplasia.
- Type 3: With IEL and crypt hyperplasia; mild (A), distinct (B), entirely flattened (C) mucosa.
- Type 4: Flat mucosa with atrophic mucosa (Crypt hypoplasia) and mild inflammation.

### Statistics

The data obtained were registered by SPSS 18.0. Frequency analyses were carried out. Continuous variables were calculated as mean ± standard deviation. In the comparison of group rates, Pearson correlation analysis was utilized in the determination of correlation between two groups and *p* < 0.05 was accepted as significant. Spearman correlation analysis was applied to age, sex, histological phase of the patients diagnosed with vitamin deficiency and their relation with *H. pylori*.

## Results

In the study, a total of 52 patients including 27 female (51.9%) and 25 male (48.1%) were evaluated as a patient group and 50 as a healthy control group involving 25 female (50%) and 25 male (50%). The average age of patients was 9 ± 4.3 years old, average weight was identified as 16.2 ± 6.3 kg. Patients’ weight and BMI values were determined signifantly low compared to those of control group. Demographical data of patient and control group were given at Table 2.

**Table 2:** The demographic data of patients and control group

	Celiac (*n* = 52)	Control (*n* = 50)	*p*
Age (Year)	9 ± 4.3	8.7 ± 5.2	0.12
Weight (kg)	16.2 ± 6.3	22.6 ± 2.8	*0.031*
Height (cm)	110.6 ± 38.5	114 ± 26.4	0.084
BMI(kg/m^2)^	16.4 ± 3.6	21.2 ± 3.4	*0.042*

*BMI* Body mass ındex

The weight of a total of 38 patients was < 2 SDS (18 female, 20 male), the height of 22 patients was < 2 SDS (10 female, 12 male). Growth retardation is the most frequent symptom determined in our patients. Abdominal pain (51.9%) and diarrhea (11.5%) were among the other most commonly seen symptoms. The symptom rates of patients were given at Table 3.

**Table 3:** The frequency of symptoms at diagnosis celiac disease

Growth retardation	61.5% (*n* = 32)
Chronic abdominal pain	51.9% (*n* = 27)
Chronic diarrhea	11.5% (*n* = 6)
Abdominal distension	7.6% (*n* = 4)
Vomiting	7.6% (*n* = 4)
Edema	3.8% (*n* = 2)
Treatment resistan iron deficiency	3.8% (*n* = 2)

In the histological analysis of the patients, Marsh 3B *n* = 23 (45.1%) was most frequently identified. In the biopsy material, however, *Helicobacter pylori* positivity was established in 29 patients (55.8%). In celiac patients, vitamin A and D levels were significantly determined low compared to those of healthy control group. No suggestive difference was established in two groups between their vitamin E and K1 levels. When their vitamin deficiencies were compared, it was identified that there was vitamin D insufficiency in 48 (92.3%) and vitamin D deficiency in 32 patients (61.5%). Vitamin A deficiency was seen in 17 celiac patients (32.7%). The one and only vitamin deficiency determined in the healthy control group was vitamin D. Vitamin D deficiency was seen in 2 patients (4%) in healthy group, vitamin D insufficiency was established in 9 patients (18%). All laboratory data of both patients and control group were given at Table 4.

**Table 4:** Laboratory findings

Celiac disease (*n* = 52)		Control(*n* = 50)	*p*
MARSH classification			
Marsh 3b	23 (45.1%)		
Marsh 3a	13 (25.5%)		
Marsh 3c	8 (15.7%)		
Marsh 2	4 (7.8%)		
Marsh 1	2 (3.9%)		
Marsh 4	2 (%.9%)		
Antikorlar			
tTG	46		
EMA	6		
*Helicobacter pylori*	29 (55.8%)		
Hemoglobin (gr/dl)	11.3 ± 1.7	12.3 ± 1.1	0.072
Albumin (gr/dl)	3.80 ± 0.80	4.0 ± 0.7	0.16
Vitamin A (ng/mL)	4.5 ± 4.1	8.8 ± 2.6	*0.0014*
Deficiency	32.7% (*n* = 17)	None	
Vitamin D (ng/mL)	19.8 ± 7.9	27.6 ± 10.4	*< 0.001*
Deficiency	61.5% (*n* = 32)	4% (*n* = 2)	*< 0.001*
Insufficiency	92.3% (*n* = 48)	18%(*n* = 9)	*< 0.001*
VitaminE (mcg/mL)	62.5 ± 30.7	71.8 ± 44.2	0.058
Deficiency	None	None	
Vitamin K (pg/ml)	713 ± 286	828 ± 220	0.066
Deficiency	None	None	

*tTG* Tissue transglutaminase, *EMA* Antiendomisium antibody

Related factors of vitamin A and D deficiency determined in patients were investigated. Nevertheless, no relationship was determined in terms of age, histological phase, *Helicobacter pylori* and other factors (Table 5).

**Table 5:** To evaluate the relationship between vitamin levels with other factors

		Vitamin A		Vitamin D		Vitamin K		Vitamin E	
		Low	Normal	Low	Normal	Low	Normal	Low	Normal
Gender	Girl	8	19	25	2	0	27	0	27
	Boy	9	16	23	2	0	25	0	25
	*p*	0.55		0.56		na		na	
Histology	Marsh 1	0	2	1	1	0	2	0	2
	Marsh 2	2	2	2	2	0	4	0	4
	Marsh 3a	6	7	8	5	0	13	0	13
	Marsh 3b	6	17	16	7	0	23	0	23
	Marsh 3c	3	5	4	4	0	8	0	8
	Marsh 4	1	1	1	1	0	2	0	2
	*p*	0.48		0.65					
H.pylori	Yes	7	22	15	12	0	27	0	27
	No	10	15	15	10	0	25	0	25
	*p*	0.14		0.77		na		na	

## Discussion

There are many factors that could lead to the deficiency of fat soluble vitamin levels. Vitamin deficiency can develop in many situations that vitamin intake, its absorption or metabolism are influenced. For example, when vitamin D deficiency is seen, not only there is vitamin D intake insufficiency, but also there are absorption reduction situations such as celiac disease, crohn disease, short bowel syndrome and moreover, inadequate sun exposure plays a key role. Vitamin D is primarily synthesized in skin. Its oral intake has less contribution to vitamin D level in the body. Vitamin D production in the skin is in direct proportion to the density of sun light to which is exposed. Therefore, vitamin D synthesis can slow down and in some cold seasons, it can stop completely [17].

In the celiac disease (CD), proximal small intestine in the most commonly involved intestine segment. As a result of this; many nutrient, vitamin and mineral absorption are destroyed. If all segments of a small intestine are involved, there can be indistinct or none malabsorption findings as a consequence of the adaptation of distal small intestine functions. This situation is particularly valid for fat and fat soluble vitamins. Because the absolute absorption place for fats is not proximal intestines. In patients whose all small intestine segments are not involved, fat and fat soluble vitamin deficiencies may not be apparent [18, 19].

The incidence of vitamin D deficiency is inversely proportional to age in celiac disease patients. The potential theories suggested regarding this; having more sunlight exposure of little children, having less adaptation to gluten-free diet as we age, performing vitamin D replacement in little children routinely and consuming more milk-dairy products can be counted [20]. Since vitamin E and A levels are affected by oral intake amount and absorption, different results have been obtained concerning their deficiencies in children with celiac disease. No deficiency has been found regarding vitamin K level in studies. Therefore there is no consensus regarding routine study of fat soluble vitamins during their diagnosis in pediatric patients. There are limited number of studies concerning this issue and study results are also different from each other. For example, in a study performed in United States of America, it was observed that lowness determination rate in fat soluble levels in newly diagnosed CD was quite limited for vitamin E in children, it was not more for vitamin D when compared to overall frequency identified in the society, on the other hand, vitamin K and A levels were normal in all patients. As a result of this study, it was not recommended that fat soluble vitamin levels be screened [21]. In a study conducted in the east part of Turkey, Topal et al. determined vitamin D deficiency higher in children newly diagnosed with celiac disease than general society, however, they did not establish a significant difference in vitamin A and E levels [22]. Unlike other studies, we determined quite high vitamin D and A deficiency in children newly diagnosed with CD. Vitamin D insufficiency in particular was identified in the majority of the patients (92.3%) and vitamin D deficiency was seen in over half of them (61.5%). Although different outcomes were obtained in studies performed in children age groups, vitamin E and vitamin D deficiency were reported to be frequently seen in patients newly diagnosed with CD in studies carried out in adults [23–26]. Therefore, routine evaluation of fat soluble vitamin levels is recommended to be evaluated in patients newly diagnosed with CD. The difference between those diagnosed at an adult age and as a child can be associated with the duration of the disease.

There is no study measuring directly vitamin K in the literature. Vitamin K deficiency was not determined in similar studies performed by measuring prothrombin time indirectly [21, 22]. We assessed vitamin K1 level directly in our study. In patients newly diagnosed with CD, vitamin K1 deficiency was not determined though vitamin K1 level was identified lower compared to that of healthy children. Similar to other studies conducted in patients newly diagnosed with CD in their childhood [21, 22], we also established vitamin E level of the patients within normal limits and parallel to healthy children.

In children diagnosed with celiac disease, vitamin A and D deficiency were identified quite high during the diagnosis compared to healthy children in our study. Vitamin D and A deficiency were determined not to have a connection with age, sex, clinical symptoms, histological symptoms and *H. pylori* positivity. Several results were attained from limited number of studies carried out in children. Results changed in different countries and even in different regions of the same country. Both in our study and in various other studies, no relationship was determined between vitamin deficiencies and histological involvement [21, 22]. All these outcomes have made us think that fat soluble vitamin levels in patients are influenced by other factors. Probably, socioeconomical living conditions and nourishment features are among these factors.

The limitations of the study include collection of data from a single center and a small sample size of patients. Future research should involve prospective multicenter trials with larger number of children from different countries, different climates and different genetic background, in order to assess the true incidence of fat-soluble vitamin deficiency in pediatric patients with celiac disease.

## Conclusions

Vitamin D and A levels were quite low and insufficiency of these vitamins were also identified frequent compared to those of healthy children. We think that routine evaluation of vitamin D and A levels of children newly diagnosed with celiac disease can be beneficial.

## Abbreviations

BMI: Body mass index
CD: Celiac disease
ELISA: Enzyme-linked immunosorbent assay
EMA: Antiendomisium antibody
IEL: Intraepithelial lymphocyte
tTG: Tissue transglutaminase
